# Relative Performance of Non-Local Cultivars and Local, Wild Populations of Switchgrass (*Panicum virgatum*) in Competition Experiments

**DOI:** 10.1371/journal.pone.0154444

**Published:** 2016-04-27

**Authors:** D. J. Palik, A. A. Snow, A. L. Stottlemyer, M. N. Miriti, E. A. Heaton

**Affiliations:** 1 Department of Evolution, Ecology, and Organismal Biology, The Ohio State University, Columbus, Ohio, United States of America; 2 Department of Agronomy, Iowa State University, Ames, Iowa, United States of America; University of Saskatchewan, CANADA

## Abstract

The possibility of increased invasiveness in cultivated varieties of native perennial species is a question of interest in biofuel risk assessment. Competitive success is a key factor in the fitness and invasive potential of perennial plants, and thus the large-scale release of high-yielding biomass cultivars warrants empirical comparisons with local conspecifics in the presence of competitors. We evaluated the performance of non-local cultivars and local wild biotypes of the tallgrass species *Panicum virgatum* L. (switchgrass) in competition experiments during two growing seasons in Ohio and Iowa. At each location, we measured growth and reproductive traits (plant height, tiller number, flowering time, aboveground biomass, and seed production) of four non-locally sourced cultivars and two locally collected wild biotypes. Plants were grown in common garden experiments under three types of competition, referred to as none, moderate (with *Schizachyrium scoparium*), and high (with *Bromus inermis*). In both states, the two “lowland” cultivars grew taller, flowered later, and produced between 2x and 7.5x more biomass and between 3x and 34x more seeds per plant than local wild biotypes, while the other two cultivars were comparable to wild biotypes in these traits. Competition did not affect relative differences among biotypes, with the exception of shoot number, which was more similar among biotypes under high competition. Insights into functional differences between cultivars and wild biotypes are crucial for developing biomass crops while mitigating the potential for invasiveness. Here, two of the four cultivars generally performed better than wild biotypes, indicating that these biotypes may pose more of a risk in terms of their ability to establish vigorous feral populations in new regions outside of their area of origin. Our results support an ongoing assessment of switchgrass cultivars developed for large-scale planting for biofuels.

## Introduction

The once extensive North American tallgrass prairie now exists mostly as small, isolated remnant prairies scattered throughout the historical range [1]. For more than 80 years, dominant grass species native to these communities have been cultivated and widely distributed. In the US, organized efforts in prairie grass cultivation began in the 1930s, following the Dust Bowl. Traditional cultivars were planted for forage and for soil and water conservation [2]. Later, efforts to improve biodiversity and ecosystem function of the tallgrass prairie expanded the use of native grass cultivars for restoration and conservation projects. One example of such widespread cultivar use is in the Conservation Reserve Program (CRP), a land conservation effort managed by the U.S. Farm Service Agency in which agricultural land is planted with grassland vegetation to improve soil and water quality and provide wildlife habitat [3].

More recently, the development of perennial grass cultivars has been geared toward their use as feedstock for biofuels. Among the anticipated benefits associated with these biomass energy crops is their high productivity on marginal land with low input requirements [4,5]. Accordingly, emphasis has been placed on developing varieties with enhanced traits related to broad environmental tolerance and high biomass yield [6,7]. However, these objectives have also raised concerns regarding the possibility that cultivating biomass crops also could promote invasiveness by selecting for traits that increase the competitive ability and fitness of cultivars outside of managed systems [8–10]. Perennial biomass crops are of particular interest because they already share a suite of traits with well-known invasive plants. For example, *Phalaris arundinacea* L., *Sorghum halepense* (L.) Pers., and *Pueraria montana* (Lour.) Merr. are perennials that exhibit rapid growth, high biomass yield, high water/nutrient use efficiency, few pest problems, and broad tolerance to abiotic conditions, and became invasive following intentional introductions [8–10]. High-yielding bioenergy crops derived from native grasses have raised fewer concerns to date, but have not been well studied in this context.

Studies of native perennial prairie grasses have revealed genetic, morphological, and/or physiological differences between cultivated and local wild seed sources, leading to enhanced physiological performance and enhanced growth of cultivars relative to wild populations [11,12]. For example, the ‘Rountree’ cultivar of *Andropogon gerardii* Vitman demonstrated enhanced growth and greater competitive ability in greenhouse experiments [12]. In other field experiments, this cultivar also displayed higher net photosynthesis, stomatal conductance and water-use efficiency [13]. Field experiments also showed that cultivated *Schizacharyium scoparium* Michx. outperformed local non-cultivated counterparts in terms of net photosynthesis and water-use efficiency, while cultivated *Sorghastrum nutans* (L.) Nash exhibited higher stomatal conductance only. In a separate study using experimental restoration plots, cultivars of *S*. *nutans* and *S*. *scoparium* showed increased early root production and greater uptake of inorganic N and soil moisture relative to non-cultivars [14]. These studies demonstrate morphological or physiological differences that could translate into greater competitive ability for cultivars over their wild counterparts.

In some situations, introduced cultivars selected for enhanced agronomic traits related to fitness could have the potential to escape managed systems and disrupt local community structure [8–10]. Competition is the most commonly proposed mechanism for explaining the impacts of invasive plants on local plant communities [15,16], and while most invasive plant studies involve non-native species, selecting for increased competitive ability of native genotypes is also recognized as a potential risk [9]. For intact tallgrass prairie habitats, new shoots from vegetative reproduction, rather than new seedling establishment, drive changes in population biomass [17], suggesting the importance of local competitive interactions. Correspondingly, dominant perennial grass species demonstrate high competitive ability found to influence plant community structure [17–19]. Differences between ecotypes can influence genetic diversity and community structure and function [20]. For native perennial grasses, intraspecific genetic variation and differential performance between cultivar and local ecotypes have been documented, as noted above, including increased vigor in cultivars that could potentially alter plant communities [12,14]. While studies using local vs. cultivar species assemblages found no effect of source population on community diversity or multiple ecosystem processes [21,22], one study from this series found that differences altered the genetics of plant communities [23]. One hypothesis for how differences could impact plant communities is through altered species interactions, or “biotic filtering,” by competitively superior genotypes [24]. Two studies show genotypic hierarchies in ecological performance that could impact plant communities in this way [25,26].

Based on previous studies that identified genetic differences between cultivar and non-cultivar populations of native perennial grasses, as well as evidence of phenotypic differences that could presumably alter competitive outcomes, common garden experiments under field conditions are needed for direct comparisons of performance under different competition treatments.

### Study system

Switchgrass (*Panicum virgatum* L.) is a warm-season bunchgrass native to North American prairies. It represents an appealing biomass crop in the US because it is native, broadly adapted to a range of abiotic conditions, and provides other environmental services including carbon sequestration, erosion control, and wildlife habitat [27]. Switchgrass is an outcrossing polyploid that displays high genetic and phenotypic diversity across its range (e.g., [28,29]). Phylogeographic studies have shown genetic diversity and population structure organized according to two generally recognized ecotypes (upland and lowland), distinct ploidy levels within ecotypes, and geographic variation [30–35]. While upland populations (octoploid and/or tetraploid) are more prevalent in mid to northern parts of the country and lowland populations (tetraploid only) in southern parts, the two ecotypes have largely overlapping distributions and the genetic variability within each ecotype is most associated with latitude [33–35]. Plants of the upland ecotype, which tend to be found in drier habitats, are generally shorter and mature earlier than lowland types [36–38].

Cultivated biotypes of switchgrass are originally derived from one or more remnant prairie populations and are developed either through random seed increases of selected plants and/or through cycles of selection and breeding. Cultivars of both ecotypes have been widely planted in the US, and varying degrees of enhanced growth, fecundity, physiological performance, resource-use efficiency and abiotic tolerance have been observed among switchgrass cultivars in agronomic field trials (e.g., [37,38]). Population-by-location interactions in field trials carried out across large geographic regions indicate that switchgrass cultivars exhibit differential local adaptation for traits including survival, flowering time, and biomass yield [34,38]. As expected, much of this variation is attributed to ecotype and latitude of origin. Notably, these studies also reveal that cultivars were not equally affected by planting location, and some cultivars are more broadly adapted outside their ecological region of origin than others [34–39].

Because prairie grass cultivars are not highly domesticated, it is often assumed that they represent natural germplasm found in wild populations. Support for this argument came after RAPD marker data were interpreted as not having distinguished cultivars from remnant prairie populations [40]. This result, along with assumptions about the short breeding history of switchgrass and limited selection cycles, led to the conclusion that switchgrass cultivars are not genetically differentiated from remnant populations, and instead represent natural variation found across large regions of adaptation. Likewise, switchgrass cultivars are often assumed to be ecologically equivalent to remnant wild populations, and are marketed as “native” seeds, with only minimal regional qualifiers related to broad regions of adaptation. However, other studies using SSR markers [32,41] and RAPDs [42] revealed clear differentiation between cultivated and wild switchgrass populations, as well as population structure within the two groups. For example, wild populations sampled from Ohio were found to be genetically distinct from cultivars ‘Blackwell’, ‘Sunburst’, and ‘Kanlow’, with ‘Kanlow’ being the most dissimilar [34].

In this study, we provide evidence for the ability of certain cultivars being developed as biofuel crops (such as the ‘Kanlow’ types) and planted outside their region of origin to outperform local native biotypes under various environmental conditions. To date, most research on the ability of switchgrass cultivars to establish feral populations has been conducted outside the native range, in California, where experimental plants fared poorly due to dry conditions (e.g., [43–45]). Also, to our knowledge, direct comparisons between wild and non-local cultivated biotypes of switchgrass are lacking, with the exception of a pilot, common garden experiment at one site in Ohio [46], [unpublished dissertation]. In that study [46], which did not include competition treatments, some cultivars (including ‘Kanlow’) grew larger than wild genotypes and exhibited high survival and seed production, suggesting that larger-scale competition experiments comparing wild and cultivated biotypes are warranted.

### Objectives

Our long-term goal is to contribute to the development of biofuel risk assessment protocols by evaluating the invasive potential of traditional and newly developed biomass cultivars. This study examines the performance and competitive ability of several non-locally sourced cultivars of switchgrass compared with wild biotypes collected from local remnant prairies. Our primary objective was to test for differences in growth, reproduction, and phenology among biotypes under different competitive conditions using common garden experiments. We hypothesized that non-local cultivars with enhanced growth traits would outperform local wild biotypes under competitive conditions in these experiments. To our knowledge, this is one of the first studies showing that a subset of biofuel cultivars developed from native species have the potential for increased fecundity and biomass production compared to local wild populations.

## Materials and Methods

### Focal biotypes and competitor species

Four non-locally sourced switchgrass cultivars (two lowland tetraploids, ‘Kanlow’ and ‘Kanlow Nebraska 1’; two upland octoploids, ‘Blackwell’ and ‘Sunburst’), and two wild populations local to each state were used in common garden experiments in Ohio and Iowa to evaluate differences in growth, flowering phenology, and fecundity under different competitive conditions. These six groups, which we refer to as the focal biotypes and describe further below, differ with respect to their geographic origin, ecotype, ploidy level, and breeding history (Table 1; S1 Fig).

**Table 1:** Summary of switchgrass biotypes in common garden competition experiments at sites in Columbus, OH, and Ames, IA (2011–2012), including the ID for each biotype.

Biotype	ID	Status	Ecotype	Origin	Cultivation history
‘Kanlow’	KL	Cultivar	Lowland	Hughes County, OK	Developed by Kansas Agricultural Experiment Station. First collected in 1957 from one site; 200 of the resulting plants were selected and allowed to cross-pollinate.
‘Kanlow Nebraska 1’	KN1	Cultivar -unreleased	Lowland	Hughes County, OK	A synthetic population based on ‘Kanlow’ genotypes selected for winter survival near Mead, NE.
‘Blackwell’	BW	Cultivar	Upland	Kay County, OK	Developed by Kansas AES. Seed was harvested in 1934 from a single plant located in a native prairie site near Blackwell, OK. The cultivar population is derived from random seed increases.
‘Sunburst’	SB	Cultivar	Upland	Union County, SD	Developed by South Dakota Agricultural Experiment Station. Plants collected from multiple native prairies in southeastern SD and open-pollinated; selections from resulting plants were used to derive a population of 800 plants (8 families).
Ohio wild population 1	OH1	Wild	-	Marion County, OH	Seeds collected in 2010 from a remnant prairie in Daughmer Bur Oak Savannah, north-central Ohio.
Ohio wild population 2	OH2	Wild	-	Erie County, OH	Seeds collected in 2010 from a remnant prairie at NASA’s Plum Brook Research Station in northern Ohio.
Iowa wild population 1	IA1	Wild	-	Story County, IA	Seeds collected in 2010 from a remnant prairie at Doolittle Prairie State Preserve.
Iowa wild population 2	IA2	Wild	-	Story County, IA	Seeds collected in 2010 from a remnant prairie near the Heart of Iowa Nature Trail in Slater, IA.

Information for released cultivars is from USDA NRCS release brochures; information on the history of the experimental cultivar KN1 is from [47].

Cultivars were chosen because of widespread use, and because traits associated with their region of origin and/or selected for during cultivation could enhance their performance relative to local biotypes. ‘Kanlow’ (KL), ‘Blackwell’ (BW), and ‘Sunburst’ (SB) were originally selected from remnant prairie populations, and are now widely planted across large portions of the US. These cultivars were developed for high leaf area ratios and vigor [47–51]. Additionally, SB was bred to be heavy-seeded and for improved winter survival [50]. BW shows high resistance to rust and other diseases [48], and KL, selected to retain green foliage late into the season, is adapted to wet conditions [49]. ‘Kanlow Nebraska 1’ (KN1) is an unreleased population of genotypes from the base cultivar ‘Kanlow’ selected for improved winter survival and minimal lodging [47]. KN1 seeds used in our experiments came from second-generation seed produced from random mating within this synthetic population, and were obtained from Dr. Kenneth Vogel of the USDA-ARS, Univ. of Nebraska. Seeds for KL, BW, and SB were purchased from Millborn Seeds Inc., SD.

Wild accessions represent pooled seed samples from >30 widely spaced plants in each population. In Ohio, wild seeds were collected from Daughmer Bur Oak Savannah (OH1) in the Eastern Plains of north-central Ohio, and from NASA’s Plum Brook Research Station (OH2) located in the northern Lake Plains region in 2010. Permission was given by the landowner for field sampling at Daughmer Prairie (privately owned at the time of sampling), and by Environmental Specialist, John Blakeman, for NASA’s Plum Brook Research Station. In Iowa, wild seeds were collected from two remnant prairies, north of Ames at Dolittle Prairie (IA1), and southwest of Ames near the Heart of Iowa Nature Trail in Slater, Iowa (IA2) in 2010. The current lessee gave permission for field sampling in this private portion of Doolittle Prairie. No permit was needed for the former railroad remnant along bike trails in Slater, IA, where Story County allows non-destructive harvesting of seeds within the prairie. No protected species were sampled from any location.

We chose little bluestem (*Schizachyrium scoparium* Michx.) and smooth brome grass (*Bromus inermis* Leyss.) as competitor species to obtain “moderate” vs. “high” levels of competition, respectively, and because both are relevant to natural and disturbed environments where switchgrass is found. Little bluestem is a warm-season, C_4_, perennial bunchgrass that often co-occurs with switchgrass in natural sites and also in seed mixes commonly sold for re-vegetation purposes in constructed prairie settings (including CRP land). This native, sub-dominant grass is shorter than switchgrass and is fairly shade-tolerant [2]. Smooth brome, which represents a stronger competitor, is a non-native, C_3_, cool-season grass that emerges early in the summer and is often planted for hay and forage [52]. It is aggressively rhizomatous and produces numerous leaves up to ~25cm long, densely arranged around the base of the plant, and a smaller number of tall flowering shoots [2]. Since its introduction into the United States from Eurasia in the late 1800s, smooth brome has escaped cultivation and become weedy in roadside areas, forests, prairies, agricultural fields, and lawns over most of the United States and Canada [52]. Its ability to invade and negatively impact native tallgrass prairie ecosystems has also been noted [53]. Seeds for the two competitor species were purchased from Millborn Seeds Inc., SD.

### Experimental design

Competitive ability has been studied in terms of both competitive response of a plant to its neighbors and competitive effect of a plant on its neighbors [54]. In our experiments, competitive ability was assessed through the response of focal plants to controlled levels of competition, as an indicator of differences in the potential for competitive success in disturbed environments [55]. For additional approaches to studying plant competitive ability, see reviews in [54–57].

**Fig 1:**
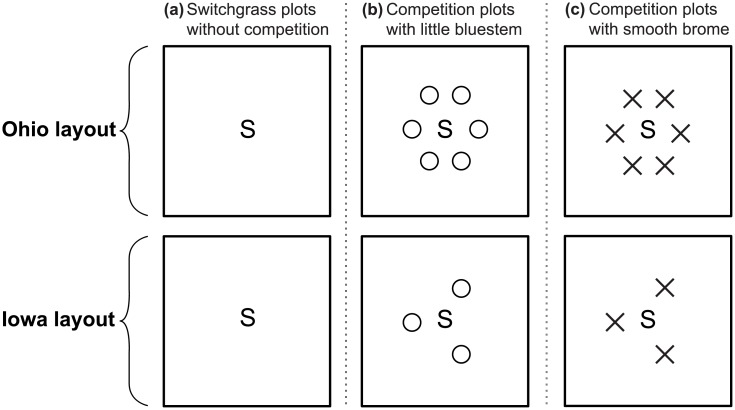
Schematic diagram of common garden experimental plots in Ohio and Iowa. Focal switchgrass biotypes were planted (a) without competition, (b) in mixture with little bluestem, or (c) in mixture with smooth brome. Competition plots contained six competitor plants in Ohio (top) and three competitor plants in Iowa (bottom).

Two common garden experiments were conducted from June 2011 to October 2012: one at Ohio State University’s Waterman Farm in Columbus, Ohio (40°00'28.4"N 83°02'09.2"W), and one at Iowa State University’s Sorenson Farm in Ames, Iowa (42°00'43.9"N 93°44'33.7"W). These were factorial experiments with a randomized complete block design with six biotypes (four cultivars and two wilds), three levels of competition (none, moderate competition from little bluestem, and high competition from weedy brome grass), and 15 replicates (18 treatment combinations x 15 replicates = 270 plots at each field site). The plots were 1.5m x 1.5m, with 1.5m buffers between plots to create a grid pattern. For the ‘no competition’ treatment plots, focal switchgrass plants were grown without competitors. In the competition plots, each focal switchgrass plant was surrounded by competitor plants arranged 30cm off-center and equidistant from each other (Fig 1). Due to a shortage of seedlings, only three competitor plants were used in Iowa, compared to six in Ohio. Despite this unplanned difference in the number of competitors, most of our results were very similar between the two locations, as described below.

### Planting methods

We soaked seeds in water overnight at 34°C before transferring them to germination boxes with moist blotting paper. At the time of germination, we planted individual seeds into 3cm peat pots filled with Fafard^®^ 2 Mix potting soil, and placed under misters in a greenhouse for approximately four weeks. Seedlings were transplanted into their respective field sites on June 20–21, 2011, in Ohio, and on June 29, 2011, in Iowa. An extra block was also planted at this time, adjacent to each field. Plants in the experimental plots that died within the first three weeks of being transplanted were replaced with seedlings from these extra blocks. Replacement transplants of focal switchgrass were minimal, occurring in six out of 270 plots in Ohio and seven out of 270 plots in Iowa (less than 3% in each case). Plots were hand-watered at the time of planting and watered twice more using sprinklers during the first month. WeedBlock^®^ fabric and mulch were used to manage weeds around plots, without restricting the growth of plants in the experiment. Uniformity within competition treatments was maintained by frequent hand weeding within plots.

### Data Collection

At both field sites, plants were monitored weekly throughout the growing season for onset of flowering (first stigmas visible). For the non-local cultivars, strongly delayed flowering times could preclude seed maturation prior to the onset of winter weather. Non-destructive growth measurements were taken at the end of the growing season in 2011 and 2012. For each switchgrass plant, maximum height was recorded as the length of the tallest shoot, including reproductive shoots, and the numbers of vegetative and reproductive shoots were counted separately. The number of vegetative shoots per plant was minimal compared to number of reproductive shoots. Therefore, separate analyses for vegetative and reproductive shoots are not reported here due to the high correlation between number of reproductive shoots and total shoot production (Pearson correlation = 0.986, *P*-value < 0.001 for Ohio; 0.998, *P*-value < 0.001 for Iowa).

At the end of the 2012 growing season, we harvested aboveground biomass and recorded the total fresh weight of each plant in the field. To estimate dry biomass, a representative subsample of approx. 200g (or the whole plant if total fresh weight was less than 200g) was also collected and weighed for each plant. The samples were oven-dried to a constant mass and the final dry weight was recorded. To calculate the total dry mass of each plant, a ratio of dry-to-fresh mass was calculated for each sample and multiplied by the total fresh weight of that plant.

To estimate the total number of seeds per focal plant, we subsampled mature panicles from each plot. First, we collected, dried, and weighed three average-sized, mature panicles from each focal plant. Then, for each of three randomly selected samples per biotype x competition treatment combination, we cleaned and weighed the seeds, and determined the average weight of 100 seeds (n = 9 groups of 100 seeds). We performed simple linear regression analyses of seed weight per panicle vs. panicle weight for each biotype separately (R^2^ values ranged from 71.3% to 96.4%). We used the resulting regression equations to estimate seed weight per panicle from panicle weight for the remaining experimental plants. We then used the estimates of seed weight per 100 seeds to convert measurements of seed weight per panicle to number of seeds per panicle. Finally, we multiplied the number of seeds per panicle by the total number of reproductive panicles produced to estimate total seed production per plant.

### Statistical analyses

Ohio and Iowa data were analyzed separately due to differences in the number of competitors used and the identities of local wild biotypes in each field experiment. All statistical analyses were performed using the program R [58]. Analyses of variance (ANOVAs) were carried out after fitting linear models (function lm), to assess the effects of biotype, competition, and their interaction on height, total number of shoots, number of reproductive shoots, aboveground biomass, time of first flowering, and seed production. Data were evaluated for outliers or missing data points and transformed if necessary to meet assumptions of normality and variance. The use of covariates other than block (early plant height, growth measurements of competitor plants) did not improve the models. Significant differences among means (*P* < 0.05) were determined using Tukey’s HSD tests.

## Results

Differences among biotypes began to emerge in the first year and were consistent with those from the second year. Here we present data from the second year, representing effects that accumulated over two growing seasons. Plant survival through the second growing season was high, which is characteristic of transplant studies, with a mean overall survival of ~99% at both field sites. Analyses were made on the whole data set as well as on a subset of the data that excluded dead plants (3 out of 270 in both Ohio and in Iowa). Results for the two data sets were comparable, and the analyses presented here included only plants that survived through the end of the experiment. Therefore, survival was not integrated into performance estimates, and results should be interpreted as effects on growth and reproduction, given that a plant survives early establishment.

Results from ANOVAs were very consistent between the two field sites (Table 2). Flowering time, plant height, aboveground biomass, and seed number were affected by biotype and competition, while shoot production also was affected by the interaction of these two factors, and seed weight was affected by biotype only. The only pattern that differed between field sites was detection of a biotype x competition interaction for seed number in Iowa and not in Ohio.

**Table 2:** Results of ANOVAs testing the effects of biotype, competition, and their interaction on measures of plant performance.

Factors	Significance levels											
	Onset of flowering		Height (cm)		Shoot # per plant		Biomass (g)		Seed # per plant		Weight of 100 seeds (g)	
	Ohio	Iowa	Ohio	Iowa	Ohio	Iowa	Ohio	Iowa	Ohio	Iowa	Ohio	Iowa
Biotype (B)	***	***	***	***	***	***	***	***	***	***	***	***
Competition (C)	***	***	***	***	***	***	***	***	***	***	ns	ns
B * C	ns	ns	ns	ns	*	**	ns	ns	ns	***	ns	ns
R-sq (%)	86.0	91.0	83.2	83.5	61.4	66.5	76.2	83.5	76.7	76.6	93.01	90.1

Analysis was conducted on log transformed biomass data, square root transformed shoot counts, and fourth root transformed seed estimates. Block was used as a random factor in all analyses.

* *P-value* < 0.05,

** *P-value* < 0.01,

*** *P-value* < 0.001

Overall, competitive effects of little bluestem on target plant growth and reproduction were smaller than that of smooth brome, supporting the use of these two species to provide distinct levels of “moderate” and “high” competition. The number and magnitude of effects from the high competition treatment were comparable between Ohio and Iowa, while fewer/smaller effects of the moderate competition treatment were observed in Iowa compared to Ohio (S1 Table), most likely due to having fewer little bluestem competitors in the Iowa plots (3 vs. 6 in Ohio). Little bluestem is a bunchgrass and did not fill in around the plots like the rhizomatous smooth brome, which also emerged earlier in the spring.

### Flowering time

**Fig 2:**
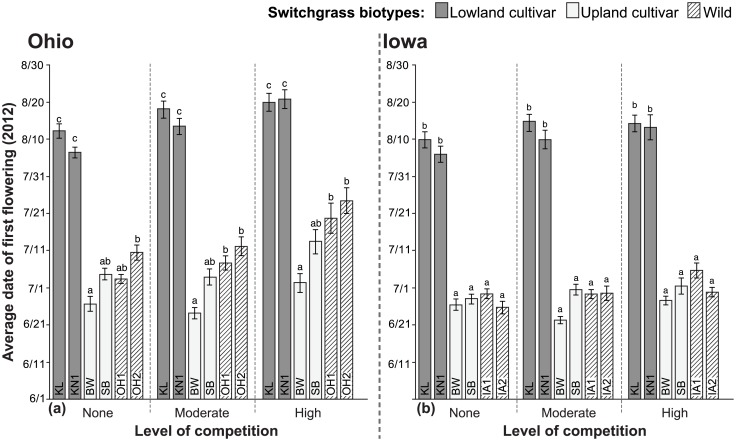
Onset of flowering in cultivated and wild switchgrass biotypes grown under three levels of competition at two locations. Date of first flowering (mean ±1 SE) was measured in Ohio (a) and in Iowa (b). Competition treatments included ‘No competition’, ‘Moderate competition’ from little bluestem, or ‘High competition’ from smooth brome. Biotypes grouped within a level of competition that share common letters are not significantly different (*P*<0.05) based on Tukey’s HSD mean comparison tests; N = 11–15. ANOVA effects are summarized in Table 2; significant differences in means among competition treatments for each biotype, based on Tukey’s HSD tests, are shown in S1 Table.

Nearly all plants successfully flowered during the second growing season (~97% overall in both Ohio and Iowa). Estimates of mean flowering onset exclude plants that failed to flower before we harvested biomass (seven plants in Ohio, six in Iowa). Omitting these plants did not alter results of statistical analyses so these data were deleted. Kanlow-type cultivars did not start flowering until later in the season compared to other biotypes, and cultivars BW and SB begin to flower earlier in the season with more similar timing to that of wild biotypes (Fig 2).

Relative differences among biotypes were largely unaffected by competition, and the only significant differences across competition treatments within a biotype were detected in Ohio, where competition significantly delayed onset of flowering of the wild biotypes as well as the cultivar KN1 by ~2 weeks (S1 Table). In Ohio, the cultivar BW had an early average onset of flowering compared to wild biotypes (~14 days earlier than OH2 with no competition and ~18 days earlier than OH1 and OH2 with competition), and the Kanlow-types started flowering 27–42 days after the Ohio wild biotypes (Fig 2a). In Iowa, KL started flowering 40–46 days later than wild biotypes while KN1 started flowering 27–37 days later than wild biotypes (Fig 2b). No significant differences were found between BW or SB cultivars and wild biotypes in Iowa, within or among competition treatments.

### Plant height

**Fig 3:**
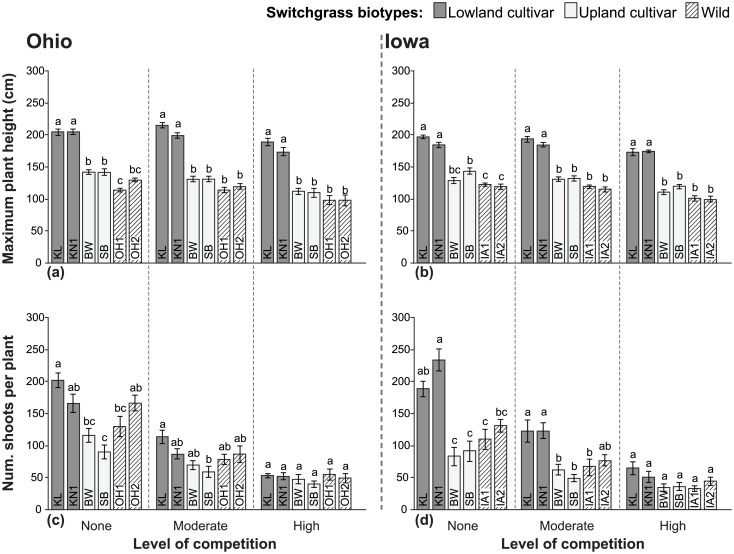
Height and number of shoots per plant for cultivated and wild switchgrass biotypes grown under three levels of competition at two locations. Mean values (±1 SE) for maximum height (a, b), and total number of shoots (c, d). Competition treatments included ‘No competition’, ‘Moderate competition’ from little bluestem, or ‘High competition’ from smooth brome. Abbreviations for biotypes correspond to those in Table 1. Biotypes grouped within a level of competition that share common letters are not significantly different (*P*<0.05) based on Tukey’s HSD mean comparison tests; N = 13–15. ANOVA effects are summarized in Table 2; significant differences in means among competition treatments for each biotype, based on Tukey’s HSD tests, are in S1 Table.

At the end of the 2-year experiment, cultivars KL and KN1 were, on average, ~1.8x as tall as the wild biotypes regardless of competition treatment (Fig 3a and 3b). In contrast, cultivars BW and SB were generally similar in height to wild biotypes. Height advantages of BW or SB over wild biotypes were detected in three comparisons, and only in plots without competition; in Ohio BW and SB averaged 1.24x as tall as OH1, and in Iowa SB was 1.18x as tall as IA1 and IA2 (Fig 3a and 3b). Height for most of the biotypes was reduced similarly by competition, with substantial decreases observed in the high competition plots compared to no competition and/or moderate competition plots, and only minimal changes between no competition and moderate competition (S1 Table). OH1 (in Ohio) and KN1 and IA2 (in Iowa) were the only biotypes with no significant differences in height among competition treatments. In terms of maximum height, KL was the most tolerant of competition with a height reduction of only 7.5% under high competition compared to no competition.

### Number of shoots per plant

Compared to plant height, the total number of shoots produced per plant was more variable. Significant differences in shoot production between cultivars and wilds were detected under no competition, with the Kanlow-types (KL and KN1) producing as many as or more shoots than the wilds, while BW and SB produced as many as or fewer than the wilds (Fig 3c and 3d). Overall, the effect of increased competition was a substantial decrease in the numbers of shoots (e.g., 56–78% fewer shoots in the high competition treatment compared to no competition), and progressively smaller differences, such that there were no significant differences between cultivated and wild biotypes under competitive conditions (see Fig 3c and 3d). One exception was observed in Iowa, where the Kanlow-types still produced 82% more shoots than IA1 under moderate levels of competition. Overall, the presence of competitors minimized differences among biotypes in shoot number, as seen in the statistical interaction between these factors (Table 2), leaving no clear advantage for one biotype over another. Because switchgrass is a bunchgrass that does not spread laterally, we would not expect differences in the numbers of shoots per plant to have much effect on persistence or competitive ability unless they are also correlated with biomass.

### Biomass

**Fig 4:**
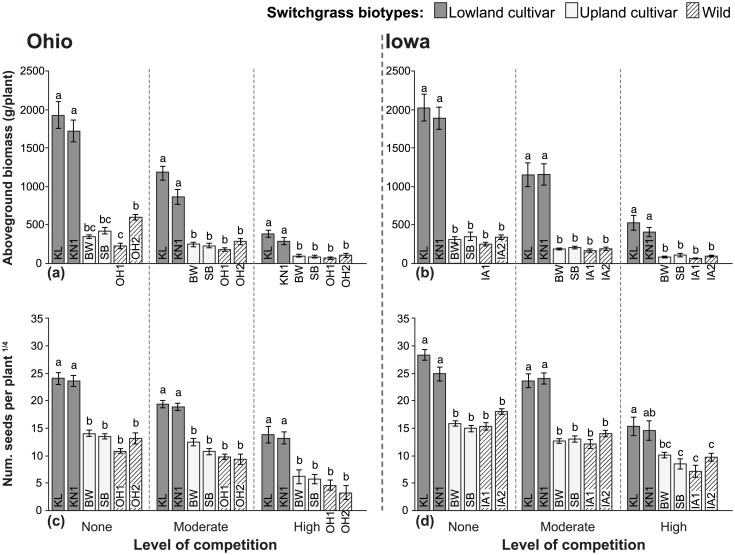
Biomass and reproductive performance of cultivated and wild switchgrass biotypes grown under three levels of competition at two locations. Mean values (±1 SE) for two fitness related traits: aboveground biomass (a, b), and number of seeds per plant (c, d). Seed production analyses included plants that survived but did not produce seed. Competition treatments included ‘No competition’, ‘Moderate competition’ from little bluestem, or ‘High competition’ from smooth brome. Abbreviations for biotypes correspond to those in Table 1. Biotypes grouped within a level of competition that share common letters are not significantly different (*P*<0.05) based on Tukey’s HSD mean comparison tests; N = 13–15. ANOVA effects are summarized in Table 2; significant differences in means among competition treatments for each biotype, based on Tukey’s HSD tests, are shown in S1 Table.

Within each competition treatment, the Kanlow-type cultivars produced at least 2x, and up to 7.5x more biomass than the corresponding wild biotypes, while no biomass differences were detected between BW or SB cultivars and the local wild biotypes (Fig 4a and 4b). The main effect of competition treatment was a substantial reduction in biomass with increasing level of competition. Reductions were largely proportional to plant size measured in plots without competition, and so the relative differences among biotypes remained consistent with increasingly competitive conditions.

### Seed production

Differences in seed production among biotypes show the same general pattern as biomass production: Kanlow-types produced substantially more seeds per plant than wild biotypes, while no significant differences were detected between BW or SB cultivars and wild biotypes (Fig 4c and 4d; note, 4^th^-root transformation of seed data is shown due to the magnitude of differences observed; see S2 Table for untransformed means and standard errors). KL produced the most seeds in both states. In plots without competition, KL averaged ~387,000 seeds per plant compared to 15,500 for OH1 and ~40,000 for OH2, and ~710,000 seeds per plant compared to ~65,000 for IA1 and ~111,000 for IA2, in Ohio and Iowa respectively (S2 Table). Compared to the magnitudes of difference in biomass, differences in the number of seeds produced were generally much greater. For example, in high competition plots, KL produced ~5x more biomass than OH1 and over 30x more seeds (Fig 4a and 4c; S2 Table). In high competition plots in Iowa, KL produced about 7.5x more biomass than IA1 and 12.5x more seeds (Fig 4b and 4d; S2 Table).

In both states, plants in the high competition plots had the lowest seed production. Overall, high competition resulted in an 84–93% decrease in seed production of biotypes in Ohio, and a 79–89% decrease in Iowa, compared to plots without competition (S1 Table). In Ohio, moderate competition also reduced seed production compared to no competition (by 28–67%). In Iowa, a small but significant interaction between competition and biotype was detected, where seed production of the cultivars BW and KN1 were not different under high competition (Fig 4d).

### Seed weight

**Fig 5:**
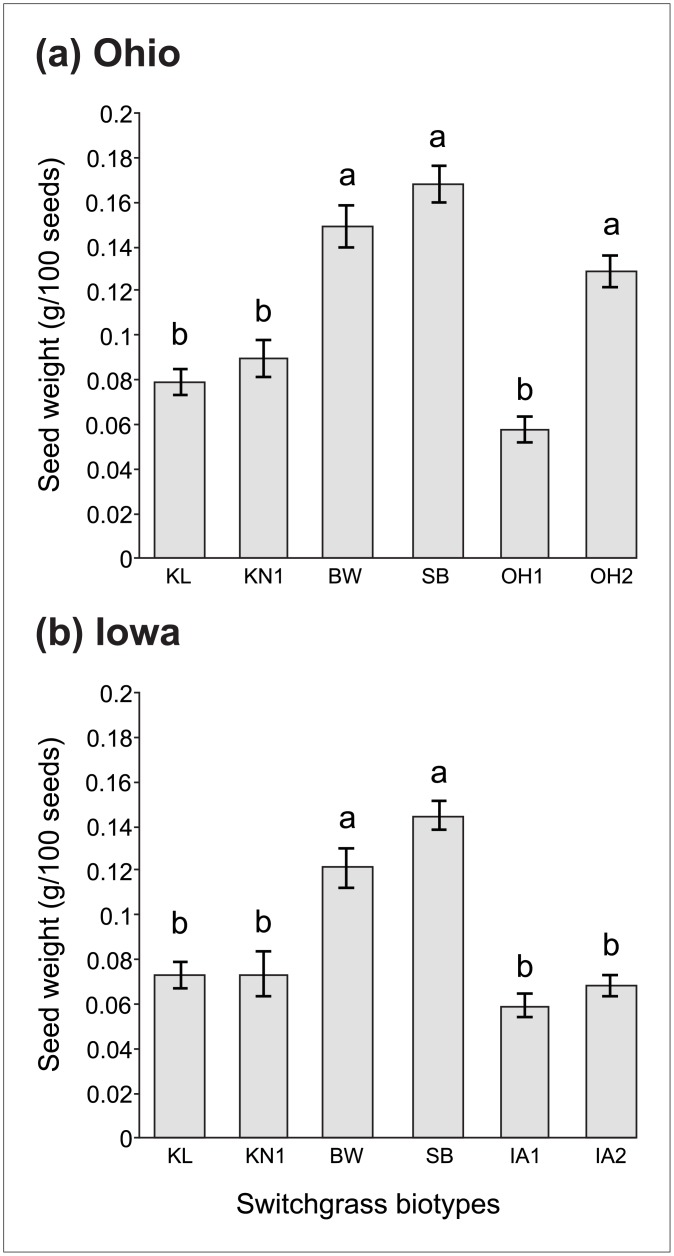
Mean weight of seeds collected from cultivated and wild switchgrass biotypes grown for 2 years at two locations. Mean weight (g per 100 seeds) ±1 SE recorded in Ohio (a) and in Iowa (b). Mean weight for each biotype is averaged across competition treatments, as this trait was insensitive to the competition treatments (non-significant effect of competition, Table 2). Abbreviations for biotypes correspond to those in Table 1. Biotypes that share common letters are not significantly different (*P*<0.05) based on Tukey’s HSD mean comparison tests; n = 9.

Biotype had a main effect on the average weight of seeds produced by plants in the common garden experiments (Table 2). Cultivars BW and SB, along with the wild biotype OH2, produced the largest seeds (Fig 5). While comparable in weight to seeds from OH2, seeds from BW and SB were 159 and 193% heavier than those produced by OH1 (Fig 5a). Overall, the Kanlow-types had smaller seeds compared to BW and SB cultivars (Fig 5). The Kanlow-type seeds were similar in size to those of wild biotypes from both states, except for being ~34% smaller than the OH2 biotype (Fig 5a). In Iowa, the larger seeds of BW and SB cultivars were ~100% heavier than seeds from wild biotypes and Kanlow-types. No significant differences in seed weight were found between KL or KN1 and Iowa wild biotypes (Fig 5b).

## Discussion

Large-scale production of cultivated switchgrass and other native grasses as biomass energy crops carries the possible risk of increasing the invasiveness of these native perennial species through increased probability of escape (via high seed production and dispersal rate) and establishment outside of planted fields [8]. The risk of cultivar escape into adjacent habitats depends on the ability of cultivars to establish outside of managed systems and impact local plant communities, a process mediated by the cultivars’ survival, competitive ability, and propagule pressure [15,59,60]. We used common garden experiments to compare the growth, fecundity, and flowering phenology of cultivated and wild biotypes, and the relative effects of competition to generate ecological data providing insight into this issue. Our results provide evidence for important differences between non-local cultivars and local wild populations that have implications for the establishment and persistence of feral populations.

A key finding of our work is that while some cultivars outperformed local biotypes others did not, suggesting that some cultivars may present more of an invasive risk than others. Specifically, evidence for greater competitive ability in KL and KN1 was found in greater aboveground biomass and also higher seed production, despite significantly later flowering times, relative to other biotypes. With competition present, these cultivars produced similar numbers of shoots as wild biotypes, but those shoots were taller and had more biomass, reflecting a greater ability to acquire resources. The greater height also could confer a competitive advantage for KL and KN1 over local wild biotypes, particularly if associated with faster growth rate early in the season.

BW and SB cultivars performed at similar levels to wild biotypes for most response variables. One possible ecological advantage was found in heavier seed weight compared to both wild biotypes in Iowa and one in Ohio. Although we did not assess associations between seed size and early fitness, previous studies with the cultivar ‘Sunburst’ reported its larger seeds were associated with greater seedling vigor when compared to other northern-adapted cultivars [50]. This is consistent with studies showing fitness benefits of larger seed size in other taxa [58–62]. Conversely, larger seeds may be less likely to disperse as far as smaller seeds, in which case this trait could also decrease the rate of dispersal. Additional studies of seed survival, dormancy, dispersal distance, and seedling establishment would be useful for evaluating the risk of invasiveness in switchgrass cultivars.

Importantly, the relative differences in performance between the cultivars and wild biotypes were consistent among competition treatments and field sites, with the exception of the number of shoots per plant, which were similar under high competition. Thus, it appears that competitive advantages of the lowland cultivars reflect intrinsic differences in biomass, height, and fecundity, and not differential responses to competition. Our results also show that some non-local cultivated biotypes that are being developed as improved biofuels may already be ecologically differentiated from wild populations in areas targeted for large-scale biofuel production. For example, southern adapted lowland types are of interest for biofuels due to greater biomass and delayed flowering when planted north of their range of origin [47]. As ‘Kanlow’ is one of the primary switchgrass populations from which biomass crops are being developed in the US, the future expectation is that improved ‘Kanlow’ cultivars will be widely planted in biomass production fields across the native range of switchgrass. Although no wild lowland populations are found in Ohio or Iowa, seed companies recommend non-local cultivars of both ecotypes for planting in these regions.

### Conclusions

Together, competitive cultivars, intense local propagule pressure, and widespread cultivation have implications for invasiveness that emphasize the importance of collecting ecological information to better inform the field of biofuel risk assessment. Our results confirm that cultivars marketed as native (such as switchgrass cultivars sold in the US) do not always perform the same as local wild populations, and early assumptions about non-invasive potential based on the “native” label may be misleading. Our studies of switchgrass in Ohio and Iowa suggest that cultivars similar to the lowland ‘Kanlow’ will exhibit fitness advantages compared to local wild populations in common environments and under various competitive scenarios, perhaps resulting in higher invasive potential. In contrast, for cultivars similar to the uplands ‘Blackwell’ or ‘Sunburst’, with no obvious competitive advantage over local wild populations but no clear disadvantage, relative fitness under various environmental conditions is expected to be similar to the wild types and the relative risk of escape and establishment will greatly depend on propagule pressure (e.g., area planted) over time, as well as their survival and available sites for seedling establishment.

Cultivars such as ‘Kanlow’ that have higher yields and seed production than local wild biotypes may be likely to present management risks under future biomass production scenarios. Therefore, assessments of the ecological risks posed by future biofuel cultivars must consider the possibility of high potential for escape [8]. Currently, switchgrass is rarely reported to be weedy or invasive in the US (but see [10]); however, further breeding efforts, genetic engineering, and an increase in acreage has the potential to foster greater ecological impacts. Based on our results for current cultivars, we hypothesize that some biomass cultivars will have higher fitness than local wild genotypes due to selection and breeding of genotypes with high biomass, high resource-use efficiency, and fast growth.

## Supporting Information

S1 Fig Geographic origins for six switchgrass biotypes. (PDF)

S1 Table Adjusted *P*-values from Tukey's HSD tests for differences in means among three levels of competition within each biotype at two locations. (PDF)

S2 Table Mean number of seeds per plant estimated for cultivated and wild switchgrass biotypes grown under three levels of competition at two locations. (PDF)
