# Supplementary material for: Relative Performance of Non-Local Cultivars and Local, Wild Populations of Switchgrass (Panicum virgatum) in Competition Experiments
Source: PLoS One. 2016 Apr 27;11(4):e0154444. doi: 10.1371/journal.pone.0154444 (PMC4847931; doi:10.1371/journal.pone.0154444)
Supplement: S1 Fig — (PDF) [file pone.0154444.s001.pdf]

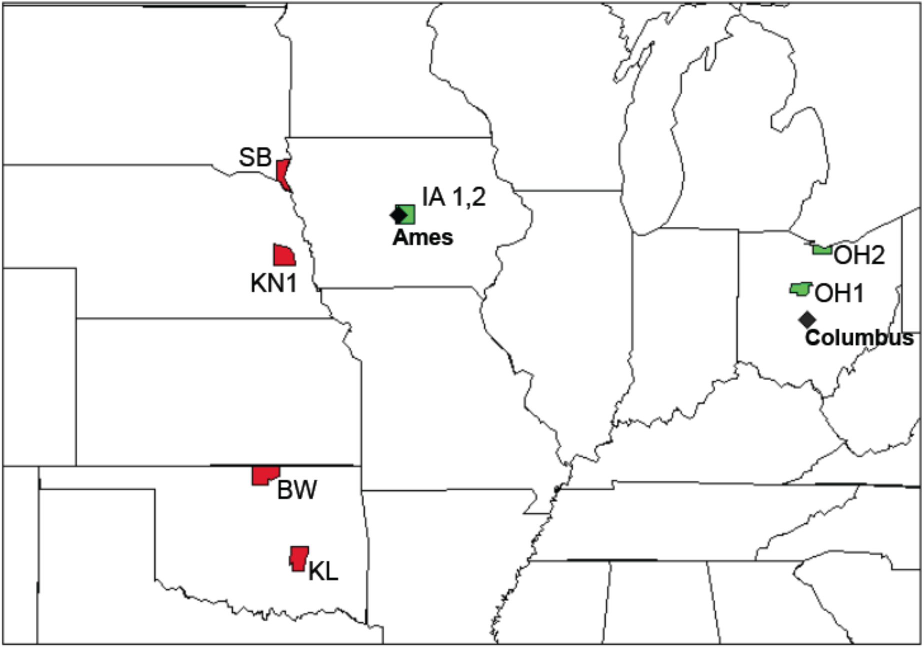

S1 Fig. Geographic origins for six switchgrass biotypes. Locations of the original source populations of three switchgrass cultivars (BW, SB, KL), sampled populations of four wild biotypes (OH1, OH2, IA1, and IA2), and two common garden field sites (Columbus, Ohio; Ames, Iowa). “KN1” indicates the location of Nebraska field station where advanced cultivar KN1 was developed from the ‘Kanlow’ base population.
