# Supplementary material for: Relative Performance of Non-Local Cultivars and Local, Wild Populations of Switchgrass (Panicum virgatum) in Competition Experiments
Source: PLoS One. 2016 Apr 27;11(4):e0154444. doi: 10.1371/journal.pone.0154444 (PMC4847931; doi:10.1371/journal.pone.0154444)
Supplement: S1 Table — (PDF) [file pone.0154444.s002.pdf]

S1 Table. Adjusted *P*-values from Tukey's HSD tests for differences in means among three levels of competition for each biotype at two locations.

		KL		KN1		BW		SB		Wild 1		Wild 2	
	**Competition**	**Moderate**	**High**	**Moderate**	**High**	**Moderate**	**High**	**Moderate**	**High**	**Moderate**	**High**	**Moderate**	**High**
Onset of flowering													
Ohio	**None**	0.9193	0.5950	0.7323	**0.0014**	1	0.9620	1	0.4398	0.9941	**0.0005**	1	**0.0044**
	**Moderate**	-	1	-	0.6883	-	0.5493	-	0.2829	-	0.0510	-	**0.0245**
Iowa	**None**	0.8905	0.9630	0.9889	0.3520	0.9763	1	1	0.9973	1	0.5339	0.9900	0.9798
	**Moderate**	-	1	-	0.9976	-	0.8260	-	1	-	0.6102	-	1
Maximum height													
Ohio	**None**	0.9805	0.7553	1	**0.0023**	0.9811	**0.0033**	0.9874	**0.0042**	1	0.7450	0.9925	**0.0019**
	**Moderate**	-	**0.0212**	-	**0.0366**	-	0.3808	-	0.3419	-	0.7356	-	0.2056
Iowa	**None**	1	**0.0052**	1	0.9729	1	0.1543	0.9178	**0.0151**	1	**0.0228**	1	0.0707
	**Moderate**	-	**0.0297**	-	0.9512	-	**0.0459**	-	0.8214	-	0.1317	-	0.3403
Shoot production													
Ohio	**None**	**0.0002**	**0.0001**	**0.0003**	**0.0001**	0.0753	**0.0001**	0.4127	**0.0029**	0.0858	**0.0001**	**0.0001**	**0.0001**
	**Moderate**	-	**0.0013**	-	0.2562	-	0.7853	-	0.9620	-	0.7960	-	0.3200
Iowa	**None**	**0.0131**	**0.0001**	**0.0001**	**0.0001**	0.9921	**0.0058**	0.1352	**0.0009**	0.2726	**0.0001**	**0.0333**	**0.0001**
	**Moderate**	-	**0.0298**	-	**0.0001**	-	0.3953	-	0.9919	-	0.2361	-	0.3674
Biomass													
Ohio	**None**	0.8735	**0.0001**	0.132	**0.0001**	0.9170	**0.0001**	0.1460	**0.0001**	1	**0.0001**	**0.0054**	**0.0001**
	**Moderate**	-	**0.0002**	-	**0.0002**	-	**0.0015**	-	**0.0005**	-	**0.0003**	-	**0.0001**
Iowa	**None**	**0.0350**	**0.0001**	0.1806	**0.0001**	0.7120	**0.0001**	0.7069	**0.0001**	0.5869	**0.0001**	0.0787	**0.0001**
	**Moderate**	-	**0.0018**	-	**0.0001**	-	**0.0001**	-	**0.0008**	-	**0.0002**	-	**0.0066**
Seed production													
Ohio	**None**	**0.0323**	**0.0001**	**0.0293**	**0.0001**	0.9983	**0.0001**	0.7364	**0.0001**	1	**0.0003**	0.1889	**0.0001**
	**Moderate**	-	**0.0017**	-	**0.0011**	-	**0.0002**	-	**0.0119**	-	**0.0084**	-	**0.0005**
Iowa	**None**	**0.0442**	**0.0001**	1	**0.0001**	0.5867	**0.0026**	0.9960	**0.0005**	0.6495	**0.0001**	0.3491	**0.0001**
	**Moderate**	-	**0.0001**	-	**0.0001**	-	0.8969	-	0.0597	-	**0.0268**	-	0.0849
