# Supplementary material for: Relative Performance of Non-Local Cultivars and Local, Wild Populations of Switchgrass (Panicum virgatum) in Competition Experiments
Source: PLoS One. 2016 Apr 27;11(4):e0154444. doi: 10.1371/journal.pone.0154444 (PMC4847931; doi:10.1371/journal.pone.0154444)
Supplement: S2 Table — (PDF) [file pone.0154444.s003.pdf]

S2 Table. Mean number of seeds per plant estimated for cultivated and wild switchgrass biotypes grown under three levels of competition at two locations.

	Ohio			Iowa		
	Biotype	Seed No.	SE	Biotype	Seed No.	SE
No competition	KL	386684.8	61506.7	KL	709531.0	83015.4
	KN1	351652.4	51710.7	KN1	458896.6	67650.9
	BW	43515.6	6031.9	BW	69082.1	8576.2
	SB	36682.8	5175.6	SB	56511.7	8659.5
	OH1	15497.0	1937.4	IA1	64626.6	12120.0
	OH2	40011.8	5515.9	IA2	111137.9	11614.3
Moderate competition	KL	158534.6	24773.3	KL	385640.0	77402.4
	KN1	141471.1	18827.3	KN1	388620.7	62560.9
	BW	28785.1	4662.3	BW	28146.9	3758.8
	SB	15782.2	2534.9	SB	33353.7	5180.2
	OH1	11091.2	2065.2	IA1	27816.4	4922.2
	OH2	13152.0	3546.1	IA2	45159.5	7004.4
High competition	KL	62386.7	10721.6	KL	97940.8	32301.1
	KN1	46506.7	10423.6	KN1	96707.9	34232.6
	BW	7045.2	2322.5	BW	13346.0	3407.5
	SB	2476.3	754.5	SB	9715.6	3350.2
	OH1	1810.8	668.2	IA1	7237.6	2201.1
	OH2	2860.9	1327.9	IA2	13172.6	3268.8
